# Primary congenital anomalies of the coronary arteries and relation to atherosclerosis: an angiographic study in Lebanon

**DOI:** 10.1186/1749-8090-4-58

**Published:** 2009-10-29

**Authors:** Ali H Eid, Ziad Itani, Mohammad Al-Tannir, Said Sayegh, Ali Samaha

**Affiliations:** 1 Department of Biology, College of Science, United Arab Emirates University, Al-Ain, UAE; 2 Department of Internal Medicine, Makassed General Hospital, Beirut, Lebanon; 3 Department of Human Morphology, Faculty of Public Health, Lebanese University, Zahle, Lebanon; 4 Cellular and Molecular Signaling Research Group, Departments of Biology and Biomedical Sciences, Faculty of Arts and Sciences, Lebanese International University, Beirut, Lebanon

## Abstract

**Background:**

Most coronary artery anomalies are congenital in origin. This study angiographically determined the prevalence of different forms of anomalous aortic origins of coronary anomalies and their anatomic variation in a selected adult Lebanese population. Correlation between these anomalies and stenotic coronary atherosclerotic disease was also investigated.

**Methods:**

4650 coronary angiographies were analyzed for anomalous aortic origin. These anomalies were clustered in four main groups: anomalous left circumflex (LCX) coronary artery, anomalous right coronary artery, anomalous left main coronary artery and anomalous left anterior descending coronary artery.

**Results:**

Thirty four patients had anomalous aortic origin of coronary arteries. Of these, anomalous LCX coronary artery was the most common (19 of 34 patients). The second most common anomaly was anomalous RCA origin (9 of 34 patients.) The incidence of coronary stenosis in non-anomalous vessels was 50%. However, a significantly smaller percentage (17.46%; 6 of 34 patients) of anomalous vessels exhibited significant stenosis, reminiscent of atherosclerotic disease. Of these six vessels, five were LCX coronary artery arising from right coronary sinus or from early branch of right coronary artery. The sixth was right coronary artery arising from left coronary sinus.

**Conclusion:**

The incidence of congenital coronary anomalies in Lebanon is similar to other populations where the most common is the LCX coronary artery. Isolated congenital coronary anomalies do not increase the risk of developing coronary stenosis or atherosclerosis. Angiographic detection of these anomalies is clinically important for coronary angioplasty or cardiac surgery.

## Background

The most common cause of sudden cardiac death in young athletes is coronary artery anomalies [1]. Primary congenital anomaly of coronary arteries is one that is not necessarily associated with any other congenital heart disease. Most coronary artery anomalies are congenital in origin owed to variation during embryonic development [2]. The term coronary artery anomaly refers to a wide range of congenital abnormalities involving the origin, course and structure of epicardial coronary arteries [3]. Although these anomalies, which are remarkably different from the normal structure, exist as early as birth, they are incidentally encountered during selective angiography [1,4,5]. These anomalies are found in 0.6-1.5% of coronary angiograms [2,5-8]. Importantly, they may predispose the patient for developing an acute myocardial damage and/or chronic injuries in the area supplied by the anomalous coronary artery originating from the incorrect coronary sinus of Valsalva [2,7,9,10].

Diagnosis and understanding of coronary artery anomalies are important in considering the severity of coronary artery stenosis, particularly during therapeutic maneuvers such as angioplasty and bypass surgery [1]. Unfortunately, no study has examined the incidence of these anomalies in the Lebanese population (around 4 million total population).

The aim of this study was to assess the prevalence of different forms of anomalous aortic origins of coronary anomalies and their anatomic variations in a selected adult Lebanese population.

## Methods

We reviewed the database of 4650 adult patients who underwent coronary angiography in cardiac catheterization unit at Makassed General Hospital in Beirut, Lebanon from April 2000 through April 2007 to determine the incidence of coronary artery anomalies. These patients had been admitted to the cardiology department: regular floor or cardiac care unit, for chest pain, palpitation, and dyspnea or effort angina. However, patients whose coronary anomalies were due to congenital heart disease, separate origin of the conus branch or right ventricular branch from the right sinus of Valsalva, coronary artery bridging, coronary arteriovenous fistulas, coronary artery aneurysms, coronary stenosis or anomalous pulmonary origin of the coronary arteries were excluded.

At least two independent investigators reviewed the films, which were selected for further assessment, prior to the final classification. In the event of any discrepancy between the two reviewers, a consensus was reached after discussion. The course of anomalous artery was defined according to the guidelines of Yamanaka and Hobbs [11] and the "eye-and-dot method" [12]. Most of the selective coronary angiographies were performed by the Judkins (femoral) method, although some were done according to the method of Sones (brachial).

In addition to demographic characteristics including age and gender, admission diagnosis was categorized as acute coronary syndrome, arrhythmia or congestive heart failure. Co-morbidities such as diabetes mellitus, hypertension and dyslipidemia were reviewed. Smoking status and family history of cardiac disease were also noted. Moreover, laboratory, electrocardiographic, cardiac angiographic results and treatment ordered were all recorded.

Electrocardiographic findings were collected either as ischemia or injury. Cardiac angiographic outcomes were described as: Left Circumflex (LCX) coronary artery arising from right coronary sinus, LCX arising as early branch of Right Coronary Artery (RCA), left anterior descending coronary artery (LAD) arising from right coronary sinus, RCA arising from left coronary sinus, and aberrant origin of left main coronary artery.

Patients were categorized as having stenosis/atheroslerosis when a significant lesion (defined as more than 50% narrowing of intraluminal diameter) was present in one or more coronary arteries or in a major branch.

### Statistical analysis

Data are presented as mean (± SD) and number (%). Chi-square test was used to assess any significant difference between types of stenosis/atherosclerosis and co-morbidities. This test was also used between stenosis/atherosclerosis and lipid profile, in addition to cardiac enzymes. Ischemia and injury detected on electrocardiograms were also tested with anomalous vessels using Chi-square test for any significant difference. *P*-values < 0.05 were considered significant.

## Results

Data of 4650 patients who underwent coronary angiography were reviewed. Thirty-four patients who had anomalous origins of coronary arteries from the aorta were entered into final data analysis. Angiography was indicated to evaluate the coronary artery disease in these patients.

The overall incidence of primary congenital coronary anomalies was 2.04% (95 out of 4650 patients) in our angiographic population. 61 patients were later excluded, as they had separate ostia for left anterior descending and LCX coronary artery arising from the left coronary sinus of Valsalva, which was considered a normal variant pattern. Thus, the true incidence of primary congenital anomalies was 0.73% (34 out of 4650 patients) of whom 26 were males (76.47%) and only 8 were females (23.53%). The mean age was 59.64 (± 13.71) years, with a range between 30 and 85 years. Additional patients' characteristics are presented in table 1.

**Table 1:** Patients characteristics (total of 34 patients)

	**Number (percentage)**
Admission diagnosis	
Acute coronary syndrome	25 (73.52%)
Arrhythmia	2 (5.88%)
Congestive heart failure	2 (5.88%)
Exclusively cardiac angiography	5 (14.70%)

Admission Unit	
Coronary Care Unit	16 (47.05%)
Regular floor	18 (52.94%)

Diabetes Mellitus	7 (20.58%)

Hypertension	20 (58.82%)

Dyslipidemia	7 (20.58%)

Smoking	17 (50.00%)

Family history of cardiac disease	
Yes	29 (85.29%)
No	5 (14.71%)

Indicative treatment	
Medical	19 (55.88%)
PTCA	9 (26.47%)
CABG	6 (17.65%)

PTCA: Percutaneous transluminal coronary angioplasty.

CABG: Coronary arteries bypass grafting.

**Figure 1:**
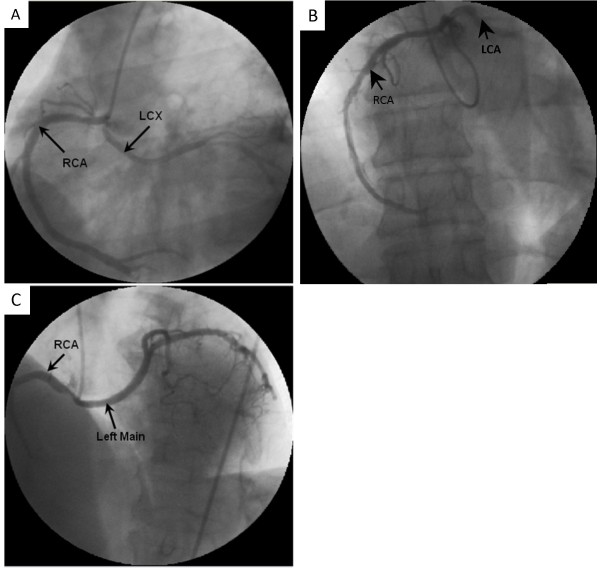
**A) Left anterior oblique view showing anomalous left circumflex (LCX) artery originating from the right coronary artery (RCA) traversing in a retroaortic course**. Note the severe coronary stenosis in its proximal part. **B) **Right anterior oblique view showing anomalous RCA originating from left coronary artery (LCA). **C) **Left anterior oblique view showing anomalous left main from RCA.

Anomalous LCX was the most common coronary anomaly being present in 19 patients (55.88%) with angiographic incidence of 0.41% (Table 2). It originated from the right sinus in three patients and from the RCA in 16 patients (Figure 1A). Its initial course was retroaortic in all cases. Peripheral distribution of the LCX artery was normal in all of them. The left anterior descending coronary artery in all of them originated from a separate ostium in the left sinus and had a normal distribution.

**Table 2:** Incidence of different congenital coronary artery anomalies in angiographic population (total of 4650 patients).

**Coronary anomaly**	**Number of patients**	**Angiographic incidence (%)**	**Anomaly incidence (%)**
Anomalous origin of LCX from RCS/RCA	19	0.41%	55.88%

Anomalous origin of RCA from LCS	9	0.19%	26.47%

Anomalous origin of LMCA	5	0.11%	14.71%

Anomalous LAD from RCS	1	0.02%	2.94%

Prevalence of all Anomalous coronary artery	34	0.73%	100%

CAD: Coronary artery disease.

Right Coronary Artery: RCA.

Left Circumflex: LCX.

LAD: Left anterior descending artery.

LMCA: Left main coronary artery.

RCS: Right coronary sinus.

LCS: Left coronary sinus.

The second most common anomaly was anomalous RCA origin and was present in nine patients (26.47%) with an angiographic incidence of 0.19% (Table 2). The artery always coursed between the aorta and the pulmonary artery. Its final distribution was normal in all cases. Moreover, the origin and distribution of the left coronary artery were also normal (Figure 1B).

Anomalous left main coronary artery from right coronary sinus was present in five patients (14.71%) with angiographic incidence of 0.11% (Figure 1C). However, anomalous LAD was present in only one patient (2.94%) with angiographic incidence of 0.02%. This anomalous left anterior descending coronary artery was originating from the right sinus and coursing anterior to the right ventricular outflow tract with normal peripheral distribution. The LCX artery was originating from the left sinus through a separate ostium with normal peripheral distribution.

The incidence of significant coronary stenosis in the 4616 patients with non-anomalous vessels was 55%. Interestingly, this percentage differed dramatically between normal and anomalous vessels in the 34 patients with coronary anomalies. Indeed, the overall incidence of significant stenosis in normal vessels was 50% (17 out of 34 patients). However, in anomalous vessels of these 17 patients, significant coronary stenotic atherosclerotic disease was detected in only 17.65% (6 of 34 total) patients. Of these six, five were angiographically defined as LCX arising from right coronary sinus or from early branch of RCA. The sixth was from RCA arising from left coronary sinus (Table 3). In none of the patients were anomalous vessels the only ones affected by stenotic formation.

**Table 3:** Incidence of atherosclerotic coronary artery disease in patients with congenital coronary artery anomalies.

	**CAD in normal coronary vessels Number (percentage)**	**Only anomalous coronary vessel with CAD Number (percentage)**	**Normal and anomalous coronary vessels with CAD Number (percentage)**
LCX arising from right coronary sinus or from early branch of RCA (n = 19)	7 (37%)	5 (26%)	7 (37%)

RCA arising from left sinus (n = 9)	6 (67%)	1 (11%)	2 (22%)

Aberrant origin of the LMCA from right coronary sinus (n = 5)	4 (80%)	0 (0%)	1 (20%)

LAD arising from right coronary sinus (n = 1)	0 (0%)	0 (0%)	1 (100%)

Total (n = 34)	17 (50%)	6 (18%)	11 (32%)

CAD: Coronary artery disease.

RCA: Right Coronary Artery.

LCX: Left Circumflex.

LAD: Left anterior descending artery.

LMCA: Left main coronary artery.

In both anomalous and normal coronaries, no significant association was found between the presence of stenotic/atherosclerotic lesion and lipid profile, cardiac enzymes or the co-morbidities (diabetes mellitus, hypertension and dyslipidemia). However, significant association was found between the ischemic changes (assessed by electrocardiogram) and stenotic/atherosclerotic coronary artery with anomalous vessels in the LCX subgroup (*p *= 0.001).

## Discussion

The overall incidence of congenital coronary anomalies was 0.73% among patients admitted primarily with diagnosis of acute coronary syndrome. This is in agreement with 0.6-1.3% incidence reported previously in different studies [2,5,6,11,13,14]. In the largest angiographic review reported by Yamanaka and Hobbs, the incidence of coronary artery anomalies in 126,595 American people was reported as 1.3% [11].

However, we did not include patients with congenital heart disease and patients with common innocuous variations in the coronary arterial pattern (separate conal artery, separate ostia for left anterior descending and LCX artery and high 'take-off' of coronary arteries). These variations have been included in a few studies [11,14] but excluded in others [13,15-17]. The most common anomaly in our series was that of LCX coronary artery which comes in accordance with some reports [15,16]. However, others report anomalous RCA as the most common one [13,17,18]. We report a 0.19% incidence of anomalous RCA in congenital coronary anomalies, which is different from other populations, being highest incidence in Indian and lowest in German populations (0.46 and 0.04%, respectively) [17]. As anomalous left anterior descending artery is one of the rarest anomalies [6], we found it only in one patient. The functional significance of this anomaly is unknown; however, it was reported to occur more commonly in association with tetralogy of Fallot [17]. Accurate identification of origin and course of anomalous coronaries is mandatory before planning coronary interventions, so that an appropriate guiding catheter, wire advancement and balloon systems may be selected [17].

Previous studies reported incidence of anomalous origin of the LCX in adults ranging from 0-1% (Table 4) [11]. The angiographic incidence of anomalous LCX was the highest (1%) in a Central European population while the overall incidence of congenital coronary anomalies was 1.3% in the same study [14]. In Japan, the angiographic incidence of anomalous LCX was the lowest (0%) whereas the overall incidence of coronary anomalies was 0.3%, with the RCA being most commonly affected [17]. In this study, we report an angiographic incidence of 0.41% for anomalous LCX, which account for a 55.88% of the overall incidence of congenital coronary anomalies. Thus, our angiographic incidence of anomalous LCX is more similar to the Asian and Turkish population than to the American and Central European populations, likely due to genetic or ethnic factors. The anomalous LCX artery always coursed posterior to the aorta to reach its normal distribution and its course was typical in all our patients. This anomaly alone causes no functional impairment of the myocardium, and it is therefore considered benign [17]. However, this anomalous artery should be recognized during coronary angiography, especially in patients with obstructive coronary artery disease or with aortic valve disease undergoing aortic valve replacement [1,10,17]. Angiographic identification of coronary anomalies prior to cardiac surgery is of considerable importance. Surgical problems can be encountered if an anomalous vessel is excluded from perfusion during cardiopulmonary bypass or if the surgeon accidentally incises this vessel [17]. Failure to recognize them can also lead to inadequate or prolonged procedures [17].

**Table 4:** Incidence of anomalous LCX coronary artery (LCX) in patients who underwent coronary angiography in different populations.

**Author**	**Number of patients**	**Incidence of anomalous LCX (%)**	**Anomalous LCX**	**Population**
Kardos *et al*	7694	1.1	83	Central European

Yamanaka & Hobbs	126595	0.8	984	American

Cieslinski *et al*	4016	0.6	26	German

Eid *et al *(this study)	4650	0.4	19	Lebanese

Ayfer Mavi *et al*	10042	0.3	27	Turkish

Garg *et al*	4100	0.3	14	Indian

Topaz *et al*	13010	0.2	22	Hispanic (& others)

Kaku *et al*	17731	0	7	Japanese

LCX: Left Circumflex.

The cardiac surgeon should be informed about the anomalous LCX artery in order to avoid accidental compression of the vessel during valve replacement [17].

Like many others, we found that the presence of an anomalous vessel does not appear to increase the chances of coronary artery disease[11,13]. Interestingly, our results show a significantly smaller incidence of coronary stenosis in anomalous versus normal vessels, which is in agreement with previous findings [13,18]. This may suggest that anomalous arteries are relatively protected from stenotic disease. However, anomalous vessels seem to develop earlier and greater atherosclerotic lesions than normal ones, but that was found exclusively in anomalous vessels arising from the right side with a retroaortic course [19]. This indicates that the origin of the anomalous vessel may be important in how early and how big the lesion develops.

We failed to find any association between co-morbidities and significant stenotic disease in normal and anomalous coronary artery vessels. While atherosclerosis plays a critical role in its development, stenosis may also be precipitated or exacerbated by other factors. Therefore, further studies are warranted to conclusively determine the relation between anomalous coronary vasculature and atherosclerosis.

Although it is the established technique for detection of coronary artery disease, coronary angiography is considered rather invasive. Currently, attention is shifting to recent advances in imaging techniques, especially those that can provide high quality measurements. Indeed, computed tomography (CT) is becoming fundamental in the detection and diagnosis of coronary artery disease [20]. Combined with perfusion imaging, coronary CT angiography would therefore allow a greater accuracy in the diagnosis of coronary artery disease [21], especially in patients with coronary anomalies.

## Conclusion

The incidence of these aberrations in Lebanon is similar to what is reported in other populations, where the most common is the LCX coronary artery. Isolated congenital coronary anomalies do not increase the risk of coronary stenosis development. In fact, it appears that anomalous vessels are less prone to getting stenotic than normal ones are. Angiographic recognition of these anomalies has an important clinical impact in coronary angioplasty or cardiac surgery, particularly in avoiding unnecessary procedures or surgical accidents.

## Competing interests

The authors declare that they have no competing interests.

## Authors' contributions

All authors collected and managed the data as well as the relevant patient information. ZI, SS and AS ensured the consent of the patients, as per the rules and regulations within the Makassed General Hopsital. ZI, SS and AS analyzed the angiograms and the patients' medical files. AHE and AS discussed the results and prepared the manuscript. AHE answered the reviewers' questions and modified the study/manuscript accordingly. All authors critically read, discussed and approved the final draft.
